# Childhood behavioral inhibition and attachment: Links to generalized anxiety disorder in young adulthood

**DOI:** 10.3389/fpsyg.2022.933213

**Published:** 2022-09-06

**Authors:** Magdalena A. Zdebik, Katherine Pascuzzo, Jean-François Bureau, Ellen Moss

**Affiliations:** ^1^Département de psychoéducation et de psychologie, Université du Québec en Outaouais, Gatineau, QC, Canada; ^2^Département de psychoéducation, Université de Sherbrooke, Sherbrooke, QC, Canada; ^3^Department of Psychology, University of Ottawa, Ottawa, ON, Canada; ^4^Département de psychologie, Université du Québec à Montréal, Montréal, QC, Canada

**Keywords:** attachment, behavioral inhibition, generalized anxiety disorder, intolerance of uncertainty, longitudinal design, temperament

## Abstract

Generalized anxiety disorder (GAD) is under-treated yet prevalent among young adults. Identifying early risk factors for GAD would contribute to its etiological model and identify potential targets for intervention. Insecure attachment patterns, specifically ambivalent and disorganized, have long been proposed as childhood risk factors for GAD. Similarly, childhood behavioral inhibition has been consistently associated with anxiety disorders in adulthood, including GAD. Intolerance of uncertainty (IU), the tendency to react negatively to uncertain situations, has also been shown to be a crucial component of GAD. Furthermore, maternal anxiety is an important feature of developmental models of anxiety including GAD. Yet, to date, no study has examined, within a comprehensive model, how attachment and behavioral inhibition in childhood, maternal anxiety in adolescence, and IU in emerging adulthood contribute to GAD in adulthood. The present study thus examines these links using a longitudinal design with 62 Canadian participants and their mothers. At age 6, participants' attachment and behavioral inhibition were assessed observationally. Maternal anxiety was measured when participants were 14 years of age. IU and GAD were assessed when participants were 21 and 23 years of age, respectively. Structural equation modeling showed that IU mediates the relationships between behavioral inhibition and GAD, while controlling for maternal anxiety. Ambivalent and disorganized-controlling attachment patterns are also indirectly associated with increased GAD symptoms via greater IU scores. Furthermore, a direct and positive effect of behaviorally disorganized attachment was found on GAD symptoms. This longitudinal study supports integrating attachment, behavioral inhibition, and IU in a model of GAD.

## Introduction

Generalized anxiety disorder (GAD), a common mental health disorder characterized by excessive worry, is frequently undertreated among adults (Robichaud et al., 2019). Individuals suffering from GAD experience reduced quality of life and notable functional impairment, and GAD is associated with high societal and economic costs due to overuse of health care services and impacts on work productivity (Wittchen, 2002; Hoffman et al., 2008). GAD is most commonly diagnosed in adulthood with the majority of cases appearing around late adolescence and early adulthood (Rogers et al., 1999; Kessler et al., 2001). However, individuals that suffer from GAD commonly describe themselves as being lifelong worriers (American Psychiatric Association, 2013). Furthermore, individuals with GAD wait on average nearly 25 years before seeking clinical help (Rapee, 1991). Hence identifying childhood and developmental risk factors associated with GAD would help develop effective early prevention and intervention programs, in order to obviate or reduce long term suffering.

Over the years, several theoretical models of GAD have been proposed (see Behar et al., 2009 for review). Three models stand out as leading to possible clues to early life factors related to GAD. Borkovec's (Borkovec, 1994; Borkovec et al., 2004) avoidance model of worry and GAD stipulates that worry is seen as an ineffective cognitive strategy to confront threatening stimuli which also leads to avoidance of the negative physical and emotional arousal triggered by the feared situation. More recently, Sibrava and Borkovec (2006) suggested that certain predispositions linked to early life experiences can affect an individual's perception of threat, such as childhood insecure attachment. An insecure attachment could lead an individual to perceive his environment as threatening but lack the emotional regulation skills to adequately respond and hence be more at risk for GAD (Cassidy, 1995; Sibrava and Borkovec, 2006; Cassidy et al., 2009). The intolerance of uncertainty model (Freeston et al., 1994; Dugas et al., 1998, 2004) stipulates that those individuals that are unable to cope with uncertain and ambiguous situations are more at risk for increased worry and hence to develop GAD. Individuals with higher levels of intolerance of uncertainty tend to react more intensely and negatively to uncertain, novel, and ambiguous situations (Dugas et al., 2004; Dugas and Robichaud, 2007), pointing to a possible physiological predisposition. A more recent model of GAD, the emotional dysregulation model (Mennin et al., 2004, 2005), stipulates that those individuals that suffer from GAD have a lower threshold for emotional activation and experience emotions more intensely, perceive emotions more negatively, have a poorer understanding of their emotions and have inadequate emotional regulation. This model highlights both physiological predispositions for heightened emotional responses and a lack of emotional regulation skills to manage responses. Taken together, these models point to potential etiological clues viewed from the perspective of a physiological vulnerability, such as a lower threshold to react to negative stimuli more intensely, as seen in some temperamental profiles such as behavioral inhibition, and inadequate emotional regulation skills, which have been associated with insecure attachment. Indeed, merging of certain models, like the intolerance of uncertainty model and the emotional dysregulation model in order to have a more complete view of GAD, has been recently suggested (Ouellet et al., 2019).

Developmental models of GAD (Rapee, 2001) have also emphasized similar variables as important in its development. Childhood factors such as the child's own characteristics and vulnerabilities including temperament (particularly behavioral inhibition), emotional dysregulation and cognitive biases related to perception of threat, parental characteristics such as parental anxiety and parent-child interactions, and environmental factors such as stressful life events that can impact an individual's sense of control, have all been identified as possible contributing factors to the development of GAD (Rapee, 2001; Newman et al., 2013). In this paper we thus focus on four factors that have been identified as important contributors to GAD: intolerance of uncertainty (cognitive bias), behavioral inhibition (temperament), insecure attachment (parent-child interaction) and maternal anxiety (familial and heritable transmission).

## IU and GAD

Intolerance to uncertainty (IU) is the tendency to perceive and react negatively to uncertainty on a behavioral, cognitive, and emotional level. Being intolerant to uncertainty can lead to long-term negative effects, since uncertain situations can be encountered daily (Dugas et al., 2004). Indeed, IU has been consistently associated with worry and anxiety in adulthood (see Dugas et al., 2004; Behar et al., 2009), increasing the risk of developing an anxiety disorder, particularly GAD. IU is thus a central precursor in the theoretical model of GAD, as it acts as a filter in ambiguous situations, leading to negative interpretations (Dugas and Robichaud, 2007; Robichaud et al., 2019). Few studies have investigated childhood risk factors contributing to IU in adulthood (for exceptions see: Tan et al., 2010; Zdebik et al., 2018) and only one has done so prospectively, linking insecure attachment and behavioral inhibition at age 6 to IU in emerging adulthood at age 21 (Zdebik et al., 2018). Since IU is highly associated with GAD, such work supports the assumptions of theoretical and developmental models of GAD that insecure attachment, as well as increased physiological responsiveness to novelty such as seen in behavioral inhibition, could pose significant risk for later GAD.

## Behavioral inhibition, IU, and GAD

Behavioral inhibition, a tendency to withdraw in the face of novelty and uncertainty, is also linked to anxiety disorders, including GAD (Svihra and Katzman, 2004; Degnan and Fox, 2007; Karevold et al., 2009; Sandstrom et al., 2020). From birth, behaviorally inhibited children respond strongly and negatively to unfamiliar, novel, or ambiguous stimuli or situations (Kagan and Snidman, 2004). Due to this physiological predisposition, they prefer to avoid uncertain circumstances at an early age. In doing so, their avoidant behaviors are reinforced, decreasing the opportunity to habituate to these situations which puts them at risk of developing an anxiety disorder (Manassis and Bradley, 1994; Lonigan and Phillips, 2001). Recent research has also linked inhibited child behaviors (i.e., low sociability and shyness) and IU in adolescence (Hawes et al., 2021) and in adulthood (Zdebik et al., 2018). Behavioral inhibition has also been associated with anxiety disorders and GAD in children and adolescence (Hudson and Dodd, 2012; Stumper et al., 2017; Sandstrom et al., 2020). However, few studies have examined associations between this temperamental profile and GAD in adulthood prospectively (Moffitt et al., 2007; Beesdo et al., 2010).

## Attachment, IU, and GAD

Problematic parent-child relationships, particularly insecure attachment, have been associated with anxiety in children and adolescents (Kerns and Brumariu, 2014 for review) as well as in adults (Dagan et al., 2020 for review), and have been specifically linked with GAD in adulthood (Eng and Heimberg, 2006; Viana and Rabian, 2008; Cassidy et al., 2009; Schimmenti and Bifulco, 2015; Newman et al., 2016). However, most longitudinal investigations were done retrospectively and very few studies have specifically examined the links between attachment and GAD in younger populations (Hale et al., 2006). Children's reactions in stressful situations depend on their interpretations and expectations of their caregiver's behaviors and responses to their needs for comfort and care (Goldberg, 2000). According to attachment theory, a child that learns that their caregiver can be relied on for comfort and for help to regulate distress in stressful or uncertain situations will develop a secure attachment (Bowlby, 1969/1982). Conversely, when the parent is inconsistent in their ability to provide support, or alternatively rejects the child's bids for proximity when confronted with a stressful or uncertain situation, the child is at risk of developing an insecure attachment (Bowlby, 1969/1982; Chorpita and Barlow, 1998). Under such conditions, children may not learn to adequately regulate their distress, leading to a sense of uncertainty and to negative interpretations of ambiguous situations (Dykas and Cassidy, 2011).

In preschool and school-aged children, secure (B), avoidant (A), ambivalent (C), disorganized-controlling (Dcont) (caregiving-type and punitive type) and behaviorally disorganized (BehD) attachment patterns have been identified (Main and Cassidy, 1988; Cassidy et al., 1992). Studies linking these attachment patterns to parental psychological wellbeing, parental sensitivity, and child outcomes have been the object of recent systematic reviews and meta-analyses (Badovinac et al., 2018, 2021; O'Neill et al., 2021). According to attachment theory, when caregivers are sensitive, warm, predictable, responsive, and accessible, children are more likely to develop a secure attachment (B) to their caregiver (Bowlby, 1969/1982; Ainsworth et al., 1978). Within a secure relationship, the caregiver comforts their child and reduces their distress in stressful situations, thus helping the child regulate their emotions and develop capacities to self-regulate (Kopp, 1989; Cassidy, 1994; Bretherton and Munholland, 1999). An avoidant attachment pattern (A) can be observed when caregivers are less sensitive, more inaccessible, and rejecting, and children minimize their dependency upon the caregiver by acting and playing autonomously (Main and Cassidy, 1988). As for children with an ambivalent attachment pattern (C), they tend to have caregivers that can be characterized as inconsistent, unpredictable, and unreliable which can lead to feelings of uncertainty and worry about parental availability in stressful situations (Main and Cassidy, 1988). These children typically show greater vulnerability and immaturity. In a disorganized (D) attachment, caregivers can be simultaneously a source of comfort and of fear and anxiety. These caregivers are known to show frightening or frightened behaviors toward the child (e.g., blank facial expressions or severe hostility), stemming from potential mental health problems, such as severe depression, or parental maltreatment (Moss et al., 2011). An inability to tolerate the uncertainty and fear related to the caregiver leads some of these children to attempt to control their environment, including their parent, in order to regulate their own anxiety through role-reversal behaviors (disorganized-controlling pattern – Dcont), where they act in either a caregiving or punitive manner toward the parent (Main and Cassidy, 1988; Solomon et al., 1995; Moss et al., 2004). Specifically, children with a controlling-caregiving attachment pattern may want to help or cheer-up their parent, whereas children with a controlling-punitive attachment can show hostile or punitive behaviors toward their parent (Cassidy et al., 1992). As for children classified with a behaviorally disorganized and/or insecure-other attachment pattern (BehD), they can display unusual, conflicting, or incomplete movements, disoriented and disordered behaviors, confusion, and apprehension with an absence of a coherent strategy to regulate comfort-seeking behavior (Main and Solomon, 1990). These children do not and cannot attempt to regain control over the uncertainty in their family environment as it may be too chaotic (Moss et al., 2011). For all the insecure attachment patterns, caregivers' behaviors fail to contribute to the child's development of adequate emotional self-regulation. In our previous work, we found that insecure attachment in childhood at age 6, specifically the ambivalent and disorganized-controlling attachment patterns, contribute to the development of IU 15 years later, in emerging adulthood (Zdebik et al., 2018). Insecure attachment characterized by inconsistent, unavailable, and unpredictable parenting or by role-reversal in the parent-child dyad has also been linked to the development of GAD in adulthood (Cassidy et al., 2009; Tan et al., 2010), however, these studies measured attachment retrospectively.

## Maternal anxiety, IU, and GAD

It has been well documented that anxiety disorders, including GAD, run in families (Noyes Jr et al., 1987; Gerull and Rapee, 2002; Hudson and Rapee, 2004; Aktar et al., 2017; Lawrence et al., 2019). Indeed, several studies documented genetic heritability of GAD from parent to child (Scherrer et al., 2000; Hettema et al., 2001). Furthermore, environmental transmission of GAD from parent to child has been associated with parental modeling of anxious behaviors, parenting characteristics, and transmission through cognitive biases such as intolerance of uncertainty (Aktar et al., 2017 for review). Accordingly, maternal anxiety should be considered as a control variable when investigating the unique contribution of child specific risk factors of GAD.

## Prospective studies of risk factors associated to GAD

Although the aforementioned risk factors have been investigated in childhood anxiety disorders in general, relatively few studies have prospectively examined the early factors that contribute to GAD in adulthood (Moreno-Peral et al., 2014 for review). As identified in developmental models of GAD, factors found to be linked to GAD in adulthood were behavioral inhibition, previous mental health problems, parenting characteristics (low warmth and caring, high overprotection and control), parental mental health problems, stressful life events including parental divorce and childhood separation events, childhood adversity (neglect, physical, and sexual abuse), neuroticism, and smoking. However, only two of the 17 studies (Clark et al., 2007; Moffitt et al., 2007) assessing GAD in adulthood identified by Moreno-Peral et al. (2014) had a childhood age at baseline with all other studies starting their assessment in adolescence or adulthood. Hence, most risk factors were assessed during adolescence and adulthood or retrospectively. In one of the studies reviewed, following over a thousand children from the age of 3 to 32 (Moffitt et al., 2007), childhood risk factors associated with GAD in adulthood included behavioral inhibition, problematic parent-child relationship (maltreatment), maternal internalizing symptoms, and low socioeconomic status. Accordingly, no study to date has examined the longitudinal contribution of childhood behavioral inhibition, childhood attachment, and IU to the development of adult GAD, while considering the confounding influence of maternal anxiety.

## Objectives

The objective of the current study is to expand on our previous work examining longitudinal prediction of IU in emerging adulthood (Zdebik et al., 2018). Specifically, we want to examine if childhood behavioral inhibition and attachment at age 6 and IU at 21 years of age directly contribute to GAD in young adulthood (at age 23), while controlling for maternal anxiety, and whether the associations between behavioral inhibition and attachment and GAD are mediated by IU. Based on previous empirical work and models of the development of anxiety, we predicted that behavioral inhibition would independently contribute to the development of GAD (Svihra and Katzman, 2004; Degnan and Fox, 2007). Insecure-ambivalent and disorganized-controlling attachment patterns are also predicted to be associated with GAD (Cassidy, 1995; Warren et al., 1997; Dugas et al., 2004). IU is predicted to be directly associated with higher levels of GAD symptoms (Dugas et al., 2004; Behar et al., 2009). Furthermore, as previously found (Zdebik et al., 2018), behavioral inhibition and insecure-ambivalent and disorganized-controlling attachment types are also predicted to be associated with IU. This study is thus an important step, extending previous findings by Zdebik et al. (2018) by testing a comprehensive model that includes both child and mother known predictors of GAD, and considering an integrative approach to temperament and childhood contexts with cognitive factors that can mediate relationships to later mental health outcomes.

## Methods

### Participants

Participants were 62 children and their mothers, representative of the general Quebec (Canada) population, taking part in an ongoing longitudinal study examining the parent-child relationship and children's socioemotional adaptation (see Moss et al., 2006). Participants were followed from early childhood to adulthood with observational measures of behavioral inhibition and attachment, sociodemographic and psychopathology symptom measures. Participants were recruited from non-profit daycares in the Montreal, Quebec area. Non-profit daycares represent more varied socioeconomic levels than private daycares. Initial recruitment was done on a voluntary basis via announcements made by daycare management to parents whose children were 4 years-old. Parents wishing to participate in the study completed a consent form and were then contacted by phone to schedule a visit for the mother and her child to the laboratory. About 50% of parents from participating daycares, whose child was in the correct age range, agreed to participate in the research. This initial time point was not included in the current study.

In the current study, of the 129 participants at Time 1 (T1, 69 girls and 60 boys), 38% of participants were lost to attrition at Time 2 (T2), the adolescent phase (T2, *N* = 80, 47 girls and 33 boys). At Time 3 (T3), 23% (*N* = 18) of participants did not complete the young adult phase (T3, *N* = 62, 40 young women and 22 young men). At the final time point (T4), another 19% (*N* = 12) was lost to attrition (T4, *N* = 50, 33 young women and 17 young men). At T4, 42% of participants still lived at home at the time of the study and 48% were in a relationship. Twenty-four percent of participants had completed a high school degree, 29% had college-level schooling, and 47% had some university-level training. *T*-tests and χ^2^ analyses of sociodemographic variables (age, sex, maternal education, family income) were conducted to compare participants lost to attrition with those remaining in the study. These analyses revealed no significant differences between T1 and T4 (all *ps* > 0.05).

At Time 1 (T1) of the present study, the sample was heterogeneous with respect to family income level (CAD in 1992) with 18% earning <$20,000, 48% earning between $20,000 and $50,000 and 34% earning over $50,000. Average maternal education at T1 was 14.9 years (SD = 2.79) with 77% having more than a high school education. Age of the 62 participants at T1 ranged between 5 and 7 years old (M = 6.14, SD = 0.99). Time 2 (T2) measures were taken 8 years later, when participants had a mean age of 13.6 years (SD = 0.59, range = 12.6–15.0 years). Seven years later, at Time 3 (T3), participants had a mean age of 21.2 years (SD = 0.81, range = 20–23 years). Approximately 2 years later, at Time 4 (T4), participants were young adults with a mean age of 23.4 years (SD = 0.93, range = 22–25 years). The final sample of 62 participants (40 girls and 22 boys) of the present study was based on having at least one variable at the last 2 time points (T3 or T4).

#### General procedure

Participants were contacted by telephone before each phase of the project. At T1 of the current study, when children were between 5 and 7 years old, participants were sent questionnaires to complete at home which were collected by the research assistants during the laboratory session. Mothers and their children were invited to the laboratory to complete a battery of measures, which included a free-play session, a separation-reunion procedure, and questionnaires. Two research assistants greeted participants, collected the completed questionnaires and explained the sequence of the visit. The dyad was invited into an unfamiliar experimental room where they were given 2 min to explore the room and toys (free play). The child's behaviors during this initial free-play session (exploration of the room and toys with the mother) were used to code behavioral inhibition. This was followed by a joint mother-child task and a 45-min separation task during which the mother left the room to fill out additional questionnaires with an experimenter and the child completed problem-solving tasks with another experimenter in the room. Preceding each mother-child reunion was a 5-min period during which the child was free to play with toys in the room. The mother then rejoined her child in the experimental room for a 5-min reunion. Following the reunion period, the mother-child dyad remained in the room for a 10-min snack time. A second separation (about 30 min) followed the snack time and was structured similarly to the first separation. It was followed by a 5-min reunion. The child's responses during the two reunions were used to code the child's attachment classification. This procedure is similar to the procedure by Main and Cassidy (1988). It was used since the children were of late preschool and early school age. The validity of this procedure for classifying attachment behavior in preschool and early school age children has been repeatedly demonstrated (Moss et al., 2004; Groh et al., 2012; Badovinac et al., 2018, 2021; O'Neill et al., 2021).

At T2, when the children were between 13 and 15 years old, adolescents and mothers filled out questionnaires at the laboratory. For mothers, questionnaires included a measure of anxiety symptoms. At T3, when participants were approximately 21 years of age, they came to the laboratory without their mothers to complete questionnaires including a measure of intolerance of uncertainty. Finally, at T4, when participants were approximately 23 years of age, they returned to the laboratory on their own to fill out questionnaires including a measure of generalized anxiety symptoms. Participants were given $20 for their participation at each phase of the study and informed written consent from all participating families was obtained at each assessment. The study was approved by the Université du Québec à Montréal and the Université du Québec en Outaouais Research Ethics Committees.

### Measures

#### Behavioral inhibition (T1)

Behavioral inhibition was measured observationally by coding child behaviors such as spontaneous vocalizations, displays of negative affect, play, and proximity to the mother in terms of frequency and length from the videotaped initial free play session at the beginning of the laboratory visit, when children were aged between 5 and 7 years old (Zdebik et al., 2018). The video segments used to code behavioral inhibition did not overlap with the video footage used to code attachment classification. Frequency or duration (in seconds) of the operationalized behaviors were divided by the total length of the duration of the free play session and standardized. Behaviors that were not observed for over 20% of the sample were coded as either present or not (0 or 1). The behavioral inhibition score was composed of the sum of reversed spontaneous vocalizations, negative affect, proximity to mother 0 to 1 meters, reversed proximity to mother 1 meter to 2 meters, reversed proximity to mother 2 meters and over, and reversed play scores, where higher scores represented higher levels of behavioral inhibition. Videotapes were coded for behavioral inhibition by the main author, who was blind to attachment classification. A second coder, trained by the main author, coded 15% of randomly selected videotapes and was blind to behavioral inhibition and attachment classification. Intraclass correlations ranged from.83 to 1.00 (all *ps* <0.001).

#### Attachment classification (T1)

The Preschool Attachment Classification System (Cassidy et al., 1992) for the 5-year-olds and the Main and Cassidy (1988) system for the 6- to 7-year-olds, were used to classify the children's reunion behaviors. Both systems use a six-category attachment coding scheme to classify children into three organized (A, B, and C) and three disorganized (D) (controlling-caregiver [Ccare], controlling-punitive [Cpuni], and behaviorally disorganized [BehD]) attachment patterns. Videotaped reunions were coded by two authors on the current paper who were blind to the participant scores on any of the other measures. Both coders were trained by R. Marvin and achieved reliability with him on a separate sample of tapes. All coding discrepancies were resolved by reviewing the tapes until consensus was achieved. Reliability for the classifications of the 5-year-old children was calculated separately from that of the 6- and 7-year-old children, which were comparable, and both indicated excellent agreement (k = 0.86 and 0.88, respectively). Overall agreement for the major classifications (A, B, C, and D) was 88% (k = 0.81), calculated on 30% of the sample. Reliability was calculated for the disorganized classification subtypes for the 14 disorganized attachment videotapes in the reliability pool, with agreement being as follows: 4/4 (100%) for Ccare, 4/5 (80%) for Cpun, and 4/6 (67%) for BehD (overall agreement for the D subtypes was thus 80%). In the current study, in order to test if disorganized-controlling and ambivalent attachment patterns are related to the development of IU and GAD, both disorganized-controlling (Dcont) subtypes were combined for analyses as they are theoretically similar in terms of role reversal and the child's expectations of their caregiver related to feeling unprotected and vulnerable (Moss et al., 2004). BehD was left as a distinct category since it was expected to lead to different outcomes than Dcont patterns (O'Connor et al., 2011). Fifty-seven percent of the sample had a secure attachment pattern (B, *N* = 35), 18 % had an avoidant attachment pattern (A, *N* = 11), 13 % had an ambivalent attachment pattern (C, *N* = 8), 7% had a D-controlling attachment pattern (Dcont, *N* = 4) and 5% had a behaviorally disorganized attachment pattern (BehD, *N* = 3).

There were no significant differences in the relative proportions of the various attachment classifications between time points (χ^2^ tests; all *ps* >0.05), indicating no differences in attrition rates. Attachment was coded into dummy variables contrasting each specified attachment group (A, C, Dcont, and BehD) to the reference secure group (B; Cohen and Cohen, 1983). In order to identify how different attachment groups (A, B, C, Dcont and BehD) may differ on sociodemographic variables, ANOVAs and χ^2^ tests were performed at T1 with participant age, sex, maternal education, and family income. Attachment groups did not differ on any of these sociodemographic variables (all *ps* > 0.05).

#### Maternal anxiety symptoms (T2)

Maternal anxiety was measured using the anxiety scale of the Symptom Checklist-90-Revised (SCL-90-R; Derogatis, 1994). This self-report 90-item questionnaire evaluates symptoms of psychopathology. Mothers rated if each symptom applied to them in the last 7 days with a scale ranging from 0 (not at all) to 4 (extremely). The anxiety scale measures symptoms such as tension, nervousness, trembling, and feelings of terror and panic. A total average anxiety score is calculated and can range from 0 to 4. As participants were from the general population, over 25% of mothers scored zero on the scale (scores ranged from 0 to 3.1 with a median score of 0.2). Therefore, the score was dichotomized and mothers scoring 0 were classified as “non-anxious” and those scoring above 0 were classified as “anxious.” The SCL-90-R demonstrates high internal consistency, and its validity and reliability have been well documented (Derogatis and Lynn, 1999). For the current study, the measure showed excellent internal consistency (α = 0.91).

#### Intolerance of uncertainty (T3)

Intolerance of uncertainty was measured using the Intolerance of Uncertainty Scale - Short Form (IUS-12; Carleton et al., 2007). This 12-item self-report questionnaire is the short form version of the original 27-item Intolerance of Uncertainty Scale (Freeston et al., 1994). Participants rated items related to uncertainty, ambiguous situations, and future events using a scale from 1 (not at all characteristic of me) to 5 (entirely characteristic of me). Items include statements such as “unforeseen events upset me greatly” and “uncertainty keeps me from living a full life.” A total score is calculated and can range from 12 to 60. Higher scores indicate higher levels of intolerance of uncertainty. The IUS-12 is comparable and highly correlated (*r* = 0.96, *p* < 0.01) to the original long form (Carleton et al., 2007; Khawaja and Yu, 2010). It has good internal consistency, convergence, and discriminant validity (Carleton et al., 2007; McEvoy and Mahoney, 2011). For the current study, the measure showed excellent internal consistency (α = 0.89).

#### Generalized anxiety disorder (T4)

Generalized anxiety symptoms were measured using the Generalized Anxiety Disorder Scale (GAD-7; Spitzer et al., 2006), a 7-item self-reported questionnaire based on the DSM-IV definition of GAD. Participants are asked to rate how often they were bothered by given symptoms during the last two weeks on a scale from 0 (not at all) to 3 (nearly everyday). Items include statements such as “feeling nervous, anxious or on edge” and “not being able to stop or control worrying.” A total score is calculated and can range from 0 to 21. Higher scores indicate higher levels of GAD symptoms. The GAD-7 has excellent internal consistency, and good test-retest reliability, and convergence and discriminant validity (Spitzer et al., 2006). For the current study, the measure showed excellent internal consistency (α = 0.84).

#### Sociodemographic questionnaires (T1-T2-T3-T4)

Sociodemographic questionnaires were completed by mothers at T1 and T2. Information relating to family income, parental education and marital status, child sex, and child age was included in the questionnaire. At T3 and T4, the young adults completed a sociodemographic questionnaire, which included items referring to income, education, living situation, and relationship status.

## Results

### Initial results

All main analyses were conducted with the 62 participants with at least one valid data point at T3 or T4. Full Information Maximum Likelihood (FIML) was used to account for missing data at T4 (*N* = 50). Correlations, ANOVAs and *t*-tests were performed with participant age, sex, maternal education, and family income in order to identify potential sociodemographic covariates related to the dependent variable, that is, GAD scores. No significant associations were identified (all *ps* >0.05: age: *r*(48) = −0.03, *p* = 0.86; sex: *t*(43) = 1.63, *p* = 0.11; maternal education: *r*(48) = 0.07; *p* = 0.63; family income: *F*_(2,47)_ = 0.96, *p* = 0.39); therefore, they were not included in subsequent analyses. Correlations between main variables are presented in Table 1. Maternal anxiety was significantly associated to GAD, with higher maternal anxiety scores being significantly associated with higher participant GAD symptoms *t*(46) = 2.27, *p* = 0.03. Hence, maternal anxiety was included in the analysis as a control variable.

**Table 1:** Main study variables: Correlations and descriptive statistics (*N* = 62).

**Variables**	**B**	**A**	**C**	**Dcont**	**BehD**	**BI**	**IU**	**GAD**
Attachment								
Secure (B vs. other)^a^	__							
Avoidant (A vs. other)^a^	−0.54**	__						
Ambivalent (C vs. other)^a^	−0.45**	−0.18	__					
Disorganized-controlling (Dcont vs. other)^a^	−0.31*	−0.12	−0.10	__				
Behaviorally disorganized (BehD vs. other)^a^	−0.26*	−0.11	−0.09	−0.06	__			
Behavioral inhibition (BI)	−0.19	0.11	0.16	0.04	−0.03	__		
Intolerance of uncertainty (IU)	−0.25	−0.07	0.33*	0.30*	−0.10	0.31*	__	
Generalized anxiety disorder (GAD)	−0.16	0.08	0.10	0.02	0.06	0.25^†^	0.45**	__
*M*						0.00	26.82	4.0
*SD*						3.26	8.74	3.55
Range						−7.15–6.36	13–53	0–19

a Attachment coded as dummy variables.

† p < 0.1,

* p < 0.05,

** p < 0.01.

### Analysis–mediation/indirect effect

A structural equation model was tested with Mplus 8.3 (Muthén and Muthén, 1998–2011) to examine longitudinal effects of behavioral inhibition and attachment (age 5–7) on GAD symptoms in young adulthood (age 23), while controlling for maternal anxiety (measured when child was age 14). The indirect effects of behavioral inhibition and attachment on GAD symptoms via IU (age 21) were also tested.

**Figure 1:**
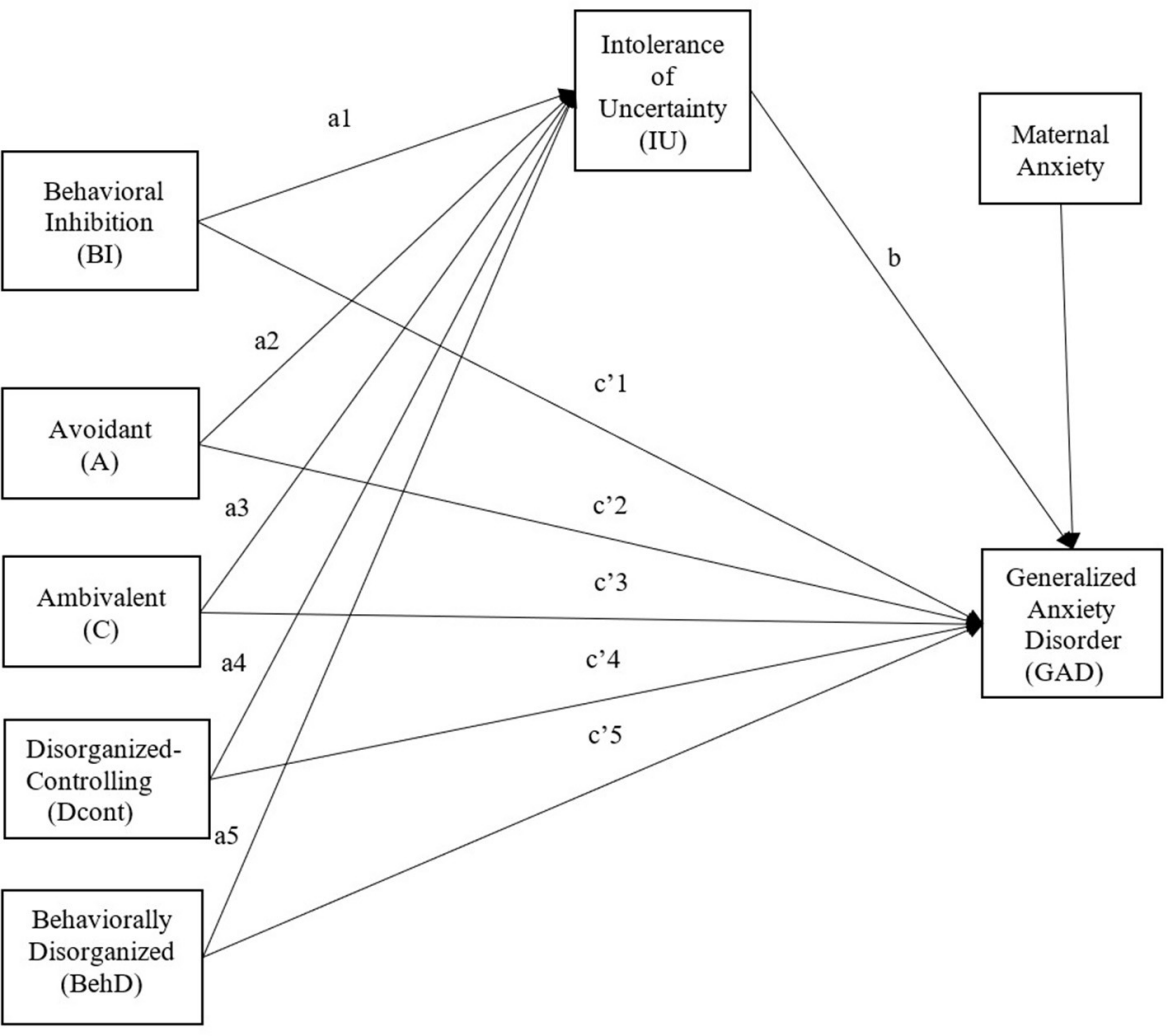
Tested model of direct and indirect effects of behavioral inhibition and attachment on GAD symptoms through IU, controlling for maternal anxiety.

First, base models were tested for direct effects of independent variables on a dependent variable and then a model was tested for indirect effects through a mediator (Figure 1). Significant indirect effects were determined using bias-corrected bootstrap confidence intervals (CI) with 2000 iterations. All models respected the usual fit indices (Hu and Bentler, 1999).

The base model for the behavioral inhibition direct effect shows that higher levels of behavioral inhibition are significantly associated with greater GAD symptoms at T4 while controlling for maternal anxiety at T2 (Table 2). We then tested for a mediation mechanism via IU. The base model examining direct effects of attachment groups on GAD while controlling for maternal anxiety (Table 3) did not reveal any significant associations. Hence, an indirect model via IU was tested.

**Table 2:** Results of the base model–Behavioral inhibition (link c).

		Fit Indices			
	Chi-Square	0.656			
	df	2			
	*p*-value	0.720			
	RMSEA	0.000			
	CFI	1.000			
		Unstandardized paths			
		b	se	*p*-value	Beta
Maternal anxiety	T3	0.98	0.37	0.01	0.28
	c1	0.25	0.11	0.03	0.22

**Table 3:** Results of the base model–attachment groups (links c).

		Fit Indices			
	Chi-Square	2.756			
	df	5			
	*p*-value	0.738			
	RMSEA	0.000			
	CFI	1.000			
		Unstandardized paths			
		b	se	*p*-value	Beta
Maternal anxiety	T3	1.10	0.37	0.03	0.31
	c2	0.93	0.87	0.29	0.10
	c3	1.36	2.22	0.54	0.13
	c4	−0.08	1.98	0.97	−0.01
	c5	1.94	2.36	0.41	0.12

The indirect model showed very good fit indices (Table 4). Results showed that behavioral inhibition and both attachment groups C and Dcont present higher levels of intolerance of uncertainty at T3 and then, increased GAD symptoms at T4 (while controlling for maternal anxiety at T2) (Table 4). Also, a direct and positive effect (*p* < 0.047) of BehD attachment was found on GAD symptoms.

**Table 4:** Results of the mediation model (links a, b and c').

		Fit Indices			
	Chi-Square	4.171			
	df	7			
	*p*-value	0.760			
	RMSEA	0.000			
	CFI	1.000			
		Unstandardized paths			
		b	se	*p*-value	Beta
Maternal anxiety	T3	0.71	0.30	0.02	0.21
	a1	0.27	0.11	0.02	0.27
	a2	−0.01	0.12	0.91	−0.01
	a3	0.36	0.12	0.00	0.35
	a4	0.30	0.12	0.01	0.30
	a5	−0.05	0.08	0.53	−0.05
	b	1.39	0.45	0.00	0.41
	c'1	0.33	0.36	0.36	0.10
	c'2	0.20	0.38	0.59	0.06
	c'3	−0.21	0.66	0.75	−0.06
	c'4	−0.41	0.47	0.37	−0.12
	c'5	0.48	0.24	0.05	0.14

Explained variances: Intolerance of Uncertainty = 31.1%; Generalized Anxiety = 23.4%.

**Figure 2:**
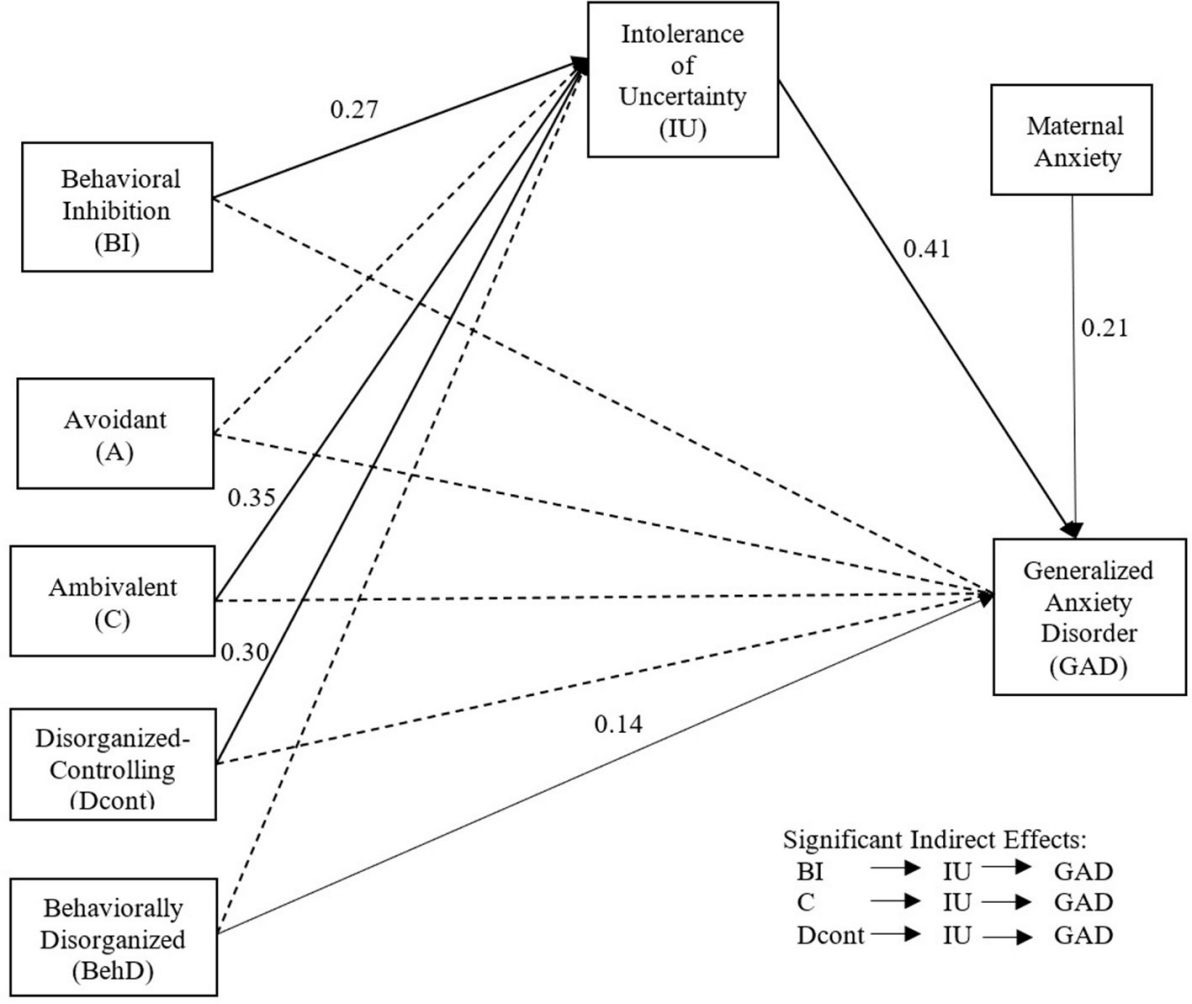
Final model of direct and indirect effects of behavioral inhibition and attachment on GAD symptoms through IU, controlling for maternal anxiety.

Table 5 presents the confidence intervals (estimated via bootstrap) of the indirect links tested between the independent variables and GAD via IU. The final model shows that IU mediates the relationship between behavioral inhibition and GAD (0 ∉ 0.066; 1.074) (Figure 2). The mediation is complete, as the direct effect of behavioral inhibition on GAD is no longer significant in the final model. Furthermore, attachment groups C (0 ∉ 0.059; 1.183) and Dcont (0 ∉ 0.089; 1.362) are also indirectly associated with increased GAD symptoms via greater IU scores.

**Table 5:** Indirect effects (Bias-corrected Bootstrap–CI).

	**Lower 2.5%**	a*b	**Upper 2.5%**
a1*b	0.066	0.379	1.074
a2*b	−0.387	−0.018	0.391
a3*b	0.059	0.499	1.183
a4*b	0.089	0.422	1.362
a5*b	−0.434	−0.072	0.144

## Discussion

While previous work has linked both childhood behavioral inhibition and attachment to IU in emerging adulthood (Zdebik et al., 2018), the longitudinal influence of these key variables for GAD in adulthood was still unknown. We thus expanded on this previous work and examined the direct contribution of behavioral inhibition and attachment in childhood (6 years old) and of IU in emerging adulthood (21 years old) to the development of GAD in young adulthood (23 years of age), while controlling for maternal anxiety. We also examined whether the associations between childhood attachment and behavioral inhibition and future GAD were mediated by IU in emerging adulthood.

As expected, results of SEM analyses revealed that IU in emerging adulthood was significantly associated with GAD symptoms in adulthood. This finding is in line with the intolerance of uncertainty model of GAD elaborated by Dugas et al. (1998), highlighting the role of IU as a main contributor to worry and GAD symptoms. Numerous studies have provided support for this model (e.g., Buhr and Dugas, 2002; Sexton et al., 2003; Koerner and Dugas, 2008), considering that individuals presenting a high level of IU are at risk of perceiving and reacting to ambiguous situations negatively. Namely, previous research has shown that individuals with higher levels of GAD symptoms report higher intolerance of uncertainty (Buhr and Dugas, 2002; Dugas et al., 2007). More recently, a meta-analysis reported that the association between IU and symptoms of GAD is significantly stronger compared to associations with IU and other disorders, such as depression, obsessive compulsive disorder, social anxiety, and eating disorders (McEvoy et al., 2019). Hence, the role of IU as a contributing and maintaining factor of GAD is undeniable (Dugas and Robichaud, 2007; Robichaud et al., 2019). While our results are in line with the intolerance of uncertainty model of GAD and previous research, it also expands on this model by integrating early risk factors of GAD, as we discuss below.

Among childhood predictors, only behavioral inhibition was directly associated with GAD over a span of 17 years, which supports previous research identifying this temperament profile as a risk factor for anxiety disorders in general in children and adults (Hudson and Dodd, 2012; Sandstrom et al., 2020) as well as for GAD specifically (Moffitt et al., 2007). Heightened negative reactions to novel or uncertain situations puts a child at risk of avoiding such situations. Over time, these avoidant behaviors, observed among children presenting high levels of behavioral inhibition, are reinforced, given their short-term appeasing effects, therefore putting the child at risk for anxiety. Having a physiological vulnerability for heightened emotional reactions to uncertain stimuli is supported by the emotional dysregulation model of GAD (Mennin et al., 2004). However, when considered in a comprehensive model including IU, results showed that behavioral inhibition was not directly related to GAD and that the association was indirect via IU. While empirical support for the association between childhood behavioral inhibition and IU in emerging adulthood has already been provided (Zdebik et al., 2018), the findings of the present study underscore the mediating role of IU in the longitudinal association between childhood behavioral inhibition and future GAD in adulthood. Indeed, behavioral inhibition has long been conceptualized as a “... vulnerability to the uncertainty caused by unfamiliar events that cannot be assimilated easily” (Reznick et al., 1989, p. 30). Behaviorally inhibited children demonstrate attentional bias toward threat, novelty, or negative stimuli and have difficulty disengaging from it (Blackford and Pine, 2012; Henderson et al., 2015). Such a cognitive bias has been proposed as a link between temperament and the development of anxiety disorders (Vasey and MacLeod, 2001; Nozadi et al., 2016). The heightened physiological reactions observed in behaviorally inhibited children could lead to a biased perception of novelty and uncertainty as threatening, increasing the risk of developing IU and eventual anxiety symptoms. Indeed, several studies found that such attentional biases, including biases against novelty, have been associated with increased risk for anxiety in behaviorally inhibited children (McDermott et al., 2009; Reeb-Sutherland et al., 2009; Lahat et al., 2014). Identifying behavioral inhibition early in a child's life would enable the implementation of prevention programs aimed at reducing heightened physiological reactions to novelty in order to prevent future intolerance to uncertainty and mental health problems (Rapee, 2013). Furthermore, individuals seeking help for GAD in adulthood that have been behaviorally inhibited as children may particularly benefit from exercises of exposure to uncertainty as treatment for their GAD (Hebert and Dugas, 2019).

Ambivalent and disorganized-controlling attachment patterns were also indirectly associated with increased GAD symptoms via greater IU scores. While previous research has shown ambivalent and disorganized-controlling attachment to be associated with an increased risk for anxiety disorders, including GAD in adulthood (Warren et al., 1997; Muris et al., 2001; Cassidy et al., 2009), we did not find such direct links. Nonetheless, the findings of the present study shed light on the underlying mechanism via IU. Indeed, children with ambivalent and disorganized-controlling attachment patterns are faced with daily uncertainty in terms of parental responses to their needs. Specifically, parents of children with ambivalent attachment are known to be inconsistent and unpredictable in their care, whereas those of children with disorganized-controlling attachment are known to be frightening or to display frightened behaviors toward their child. To gain access to their parent and minimize this uncertainty, these children have developed maladaptive socio-emotional patterns. Ambivalent children exaggerate their signals of distress to ensure their parent's responses whereas disorganized-controlling children adopt role reversal behaviors by which they take on the role of their parent (Moss et al., 2011). It is thought that taking control over the relationship is an attempt to regulate internal states such as feeling helpless and to gain control over their environment and prevent the parent from being frightening or frightened (George and Solomon, 2008). The findings of the present study suggest that over time, these children are at risk of developing a greater intolerance to uncertainty, subsequently increasing their risk of developing GAD symptoms.

Finally, a direct and positive effect of behaviorally disorganized attachment was found on GAD symptoms. A body of empirical work suggests that a behaviorally disorganized attachment pattern in the preschool years may stem from chaotic family environments in which, contrary to children with a disorganized-controlling attachment pattern, children are incapable of taking control of the situation and their environment (Moss et al., 2011). O'Connor et al. (2011) compared the disorganized-controlling and behaviorally disorganized groups in the NICHD-SECCYD sample (n = 1,364) at age 3 and found that the behaviorally disorganized group was associated with poorer outcomes than the disorganized-controlling subtypes on all of the 18 variables assessed in the study, covering maternal psychological symptoms (e.g., depression, stress), mother-child interaction (e.g., maternal hostility, lack of support) and child social adaptation (e.g., disruptive, internalizing, and externalizing behaviors). Moreover, in a small prospective longitudinal study of families at high socioeconomic risk, Bureau et al. (2009) showed that disorganized-controlling patterns in middle childhood were predicted by either maternal withdrawal (controlling-caregiving subtype) or maternal disrupted communication (controlling-punitive subtype) in infancy. In comparison, continued signs of disorganization and fear in middle childhood were associated with more severe factors such as violent and chaotic family patterns in infancy as well as maternal reports of partner physical abuse and severe physical abuse of the child. Thus, as these children presumably experience fear and anxiety on a regular basis, such an unpredictable environment can cause severe difficulties in emotional and stress regulation. Indeed, research has shown that children that experience maltreatment and bullying are at greater risk of later GAD (Copeland et al., 2013; Lakhdir et al., 2021). One striking difference between GAD and other anxiety disorders is that individuals with GAD have a large number of worries related to everyday life as opposed to specific ones (Dugas et al., 1998). Behaviorally disorganized children may hence be prone to worry more diffusely about everything in general as these children experience fear and anxiety on a regular basis which can be related to common daily life.

Taken together, the present study provides important insights into the longitudinal influences of childhood attachment and behavioral inhibition on IU, and how IU then influences GAD in early adulthood. These results are further strengthened by the fact that maternal anxiety symptoms were controlled for, since this has been repeatedly shown as an important contributor to offspring anxiety (Lawrence et al., 2019). Hence, our results support an integrative approach to GAD, one that incorporates certain aspects of prominent theoretical models of GAD, such as the intolerance of uncertainty model, the emotional dysregulation model, and the avoidance model of GAD, thus facilitating a more complete view of the development and maintenance of this disorder (Dugas et al., 2004; Mennin et al., 2004, 2005; Sibrava and Borkovec, 2006).

### Limitations and future directions

Despite the new insights our study provides, it has some limitations. First, our sample is small as it has suffered from attrition due to its longitudinal design. Attrition usually diminishes statistical power, yet we detected significant associations between variables. Still, replication in other larger populations would be beneficial. Second, attachment and behavioral inhibition were assessed using the video footage collected at the same time point of the longitudinal study creating a potential for shared method variance. However, different segments of the laboratory sessions were used to code each measure and no relation was found between the two variables, hence reducing the possibility of shared variance. Also, since behavioral inhibition was correlated with GAD while attachment was not, shared variance cannot fully account for our findings. Third, maternal anxiety was measured when participants were adolescents, meaning we were unable to control for maternal anxiety symptoms when participants were children (at age 6) or when they were older (young adulthood). Future studies should include maternal and paternal anxiety symptoms at these key developmental periods to further control the potential effects of parental anxiety in the development of GAD. Furthermore, although this is a longitudinal study, we cannot infer causality between our variables. However, our results are in line with the theoretical models of GAD, where it is widely proposed that a temperamental vulnerability and insecure attachment could have long-term effects on socio-emotional adaptation. Still, it would be important to replicate these findings in a larger longitudinal study with repeated measures from childhood to adulthood of all the main variables (attachment, temperament, child and parent anxiety) in order to better understand the temporal relationships between them. This would allow examination of the longitudinal influence of attachment and temperament from childhood to adulthood on anxiety symptoms at different stages of life. Lastly, considering the known influence of stressful life events in the development of GAD (Moffitt et al., 2007; Kessler et al., 2008; Beesdo et al., 2010), the addition of a stress indicator could extend the identified model and provide additional information on the unique contributions of attachment, behavioral inhibition, and IU in the etiology of GAD. Nevertheless, the integrative life span approach of the study strengthens the presented model.

### Conclusion and implications for practice

In sum, the findings of the current study expand the existing body of literature on the etiology of GAD by providing a clearer understanding of the direct and indirect associations between childhood behavioral inhibition and attachment, intolerance of uncertainty in emerging adulthood and GAD in young adulthood. The prospective longitudinal design and SEM statistical approach strengthen the robustness of the study. This study highlights the importance of identifying behavioral inhibition and certain types of attachment early on to reduce future risk for GAD. A particularly interesting finding is the indirect effect of IU, emphasizing that treating IU may be a key method to consider for preventing GAD among children presenting insecure ambivalent and disorganized-controlling attachment and those with high behavioral inhibition. Still, our results highlight the relevance of early and direct preventative interventions aimed at increasing attachment security and reducing behavioral inhibition in order to reduce future risk of psychopathology (Bakermans-Kranenburg et al., 2003, 2005; Mountain et al., 2017) as well as integrative interventions for current psychopathology (Chigwedere and Moran, 2022). Furthermore, the direct association, across a 17-year period, between behaviorally disorganized attachment in childhood and GAD in adulthood, is particularly striking. For these children, early interventions aiming to promote security within the parent-child relationship is especially crucial to ensure their emotional developmental and future mental health.

Since young adulthood is a developmental period particularly marked by important changes and uncertainty (e.g., important decisions, start of graduate studies, entering the work force, developing long-term relationships, moving out on one's own, etc.), learning to adequately cope with uncertainty and the potential stresses that accompany these monumental life events is crucial for promoting the well-being and mental health of young adults. Hence, strategies to help tolerate uncertainty would be of importance in emerging adulthood, but preventative measures to help with the precursors of intolerance of uncertainty and GAD, with interventions targeting behavioral inhibition and attachment, would be important avenues to pursue.

## Data availability statement

The datasets presented in this article are not readily available due to participant confidentiality. Requests to access the datasets should be directed to EM (sheiner-moss.ellen@uqam.ca).

## Ethics statement

The study was approved by the Université du Québec à Montréal and the Université du Québec en Outaouais Research Ethics Committees. Written informed consent to participate in this study was provided either by the participants' legal guardian/next of kin or the participants themselves.

## Author contributions

MZ and KP contributed to conception and design of the study. EM is the senior researcher who launched the longitudinal cohort study. MZ wrote the first draft of the manuscript. MZ, KP, and J-FB wrote sections of the manuscript. MZ, KP, J-FB, and EM reviewed the manuscript. All authors contributed to the article and approved the submitted version.

## Funding

This research was supported by the Quebec Culture and Society Research Fund (2022-NP-297325), Canada's Social Science and Humanities Research Council, the Université du Québec en Outaouais, and the Université du Québec à Montréal.

## Conflict of interest

The authors declare that the research was conducted in the absence of any commercial or financial relationships that could be construed as a potential conflict of interest.

## Publisher's note

All claims expressed in this article are solely those of the authors and do not necessarily represent those of their affiliated organizations, or those of the publisher, the editors and the reviewers. Any product that may be evaluated in this article, or claim that may be made by its manufacturer, is not guaranteed or endorsed by the publisher.
